# Elucidation of Substrate Specificity in *Aspergillus nidulans* UDP-Galactose-4-Epimerase

**DOI:** 10.1371/journal.pone.0076803

**Published:** 2013-10-07

**Authors:** Sean A. Dalrymple, John Ko, Inder Sheoran, Susan G. W. Kaminskyj, David A. R. Sanders

**Affiliations:** 1 Department of Chemistry, University of Saskatchewan, Saskatoon, Saskatchewan, Canada; 2 Department of Biology, University of Saskatchewan, Saskatoon, Saskatchewan, Canada; Instituto de Tecnologica Química e Biológica, UNL, Portugal

## Abstract

The frequency of invasive fungal infections has rapidly increased in recent years. Current clinical treatments are experiencing decreased potency due to severe host toxicity and the emergence of fungal drug resistance. As such, new targets and their corresponding synthetic pathways need to be explored for drug development purposes. In this context, galactofuranose residues, which are employed in fungal cell wall construction, but are notably absent in animals, represent an appealing target. Herein we present the structural and biochemical characterization of UDP-galactose-4-epimerase from *Aspergillus nidulans* which produces the precursor UDP-galactopyranose required for galactofuranose synthesis. Examination of the structural model revealed both NAD^+^ and UDP-glucopyranose were bound within the active site cleft in a near identical fashion to that found in the Human epimerase. Mutational studies on the conserved catalytic motif support a similar mechanism to that established for the Human counterpart is likely operational within the *A. nidulans* epimerase. While the *K*_m_ and *k*_cat_ for the enzyme were determined to be 0.11 mM and 12.8 s^-1^, respectively, a single point mutation, namely L320C, activated the enzyme towards larger *N*-acetylated substrates. Docking studies designed to probe active site affinity corroborate the experimentally determined activity profiles and support the kinetic inhibition results.

## Introduction

Pathogenic fungi, namely *Candida* and *Aspergillus* spp. [1,2], have gained notoriety in recent decades for causing life-threatening diseases within immunocompromised individuals [3], but can also pose a threat to otherwise healthy individuals [4]. *Aspergillus fumigatus*, a ubiquitous saprophytic fungus which forms airborne spores, is the predominant human fungal pathogen on account of its enhanced virulence and external environmental prevalence [5]. The staggering increase in frequency, over 200% in recent years, of invasive fungal infections [6] and associated economic and public health costs, which range in the billions (USD) per year, along with alarmingly high mortality rates are certainly cause for concern [7,8]. In this context, it is perhaps surprising to learn that the current arsenal of clinically employed antifungal drugs targets only a limited number of fungal cellular processes [9].

Many challenges for effective treatment of fungal pathogens within human hosts exist on account of the close evolutionary relationship between the eukaryotic systems [10]. As such, the potency of many antifungal drugs is severely limited due to the resulting toxicity experienced by the host during treatment [11]. Additionally, and perhaps of more concern, is that current treatments are experiencing a diminished efficacy in killing these pathogens due to the emergence of fungal drug resistance [12]. Currently, antifungal drugs which disrupt integrity and induce cell wall stress, namely the echinocandins [13,14], are believed to be the most promising candidates for clinical treatment. Perhaps not surprisingly, a mechanism for echinocandin resistance has recently been reported via a mutation within the gene encoding the catalytic subunit required for cell wall synthesis [15].

**Figure 1:**
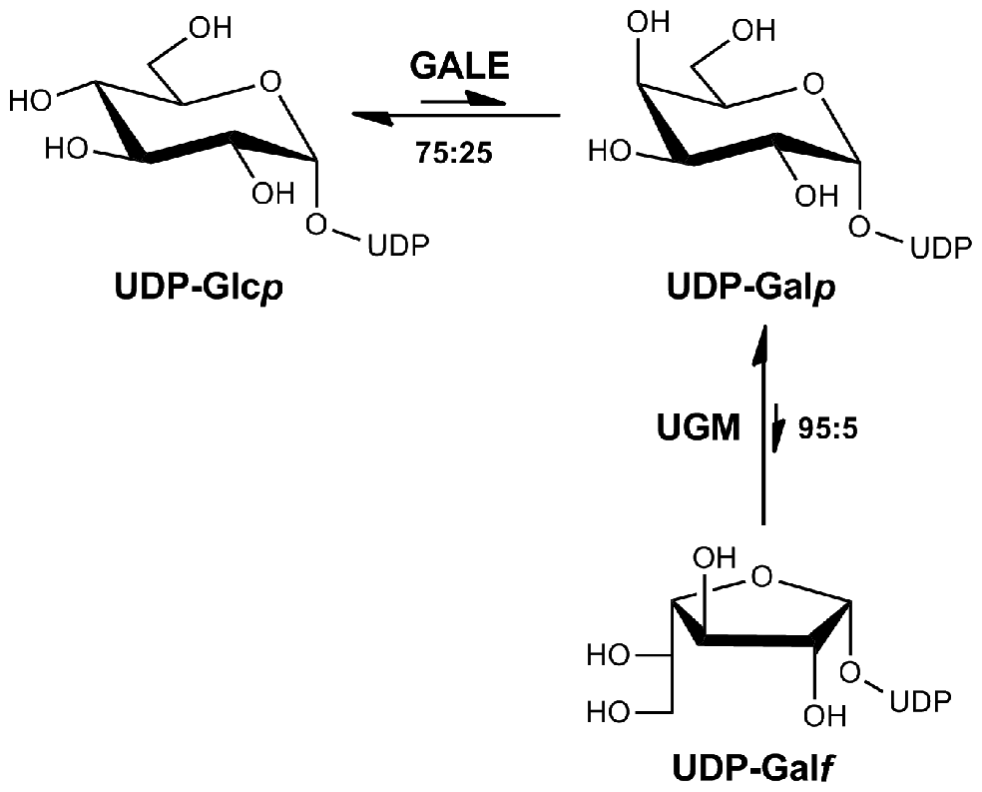
Proposed biosynthetic pathway of UDP-galactofuranose (UDP-Gal*f*) in *Aspergillus nidulans*.

The fungal cell wall, which comprises about 20% of its biomass, is responsible for mediating interactions between the fungal pathogen and the surrounding environment [16]. In the case of pathogenic *A. fumigatus*, successful host invasion relies on the coordinated expression of numerous genes involved in fungal growth, including conidial germination, cell wall assembly, hyphal growth, nutrient acquisition, and resistance to adverse conditions [17]. The composition of the fungal cell wall represents a critical interface for the host and adaptive immune responses such as remodelling of the cell wall are required for survival [18,19]. While the cell walls of *A. fumigatus* and *Aspergillus nidulans* have similar but not identical carbohydrate composition [18,20], *A. nidulans* serves as an excellent model system for studying pathogenic eukaryotic species. Importantly, some of the building blocks of the fungal cell wall, namely extracellular carbohydrates, are not found within animal systems, and as a result, they and their biosynthetic pathways are viewed as potential drug development targets [21]. Galactofuranose (Gal*f*), which is the five-membered ring form of galactopyranose (Gal*p*), is found in the walls and extracellular carbohydrate sheaths of bacteria, protists, fungi, and plants, but not in animals [22]. UDP-Gal*f* residues are directly produced from UDP-galactopyranose (UDP-Gal*p*) by UDP-galactopyranose mutase (UGM) before being incorporated into extracellular carbohydrate-containing compounds (Figure 1) [23]. Although Gal*f* is not essential within fungi, it is important for wild-type fungal growth, cell morphogenesis, wall architecture, and conidiation [24-27], as well as, pathogenesis [28-31]. In this sense, Gal*f* and its biosynthetic pathway can be viewed as potential drug targets for combination fungal therapy. As an example, in comparative studies with the wild-type strain, deletion of *A. nidulans* UGM (UGMΔ) resulted in compact colonial growth, abnormal hyphal wall structure, and reduced conidiation [25].

In a variety of species, ranging from *Escherichia coli* (*E. coli*) to Human, UDP-galactose-4-epimerase (GALE; EC 5.1.3.2), otherwise known as UDP-glucose-4-epimerase, catalyzes the interconversion of the 4'-hydroxyl configuration (Figure 1) between UDP-glucopyranose (UDP-Glc*p*) and UDP-Gal*p* [32]. As this step provides the precursor building blocks, namely UDP-Gal*p*, for the production of Gal*f* residues used in fungal cell wall assembly, GALE can also be viewed as a potential drug development target. While GALE exhibits interspecies variation at both the structural and functional level, differences between GALE of the host and pathogen can be potentially targeted for rational drug design. In this context, our laboratory has been conducting biochemical and structural studies on GALE from *A. nidulans* (AnGALE) with the goal of elucidating the structure-function relationship responsible for its catalytic role in fungal extracellular carbohydrate synthesis [33]. Initially, the gene encoding AnGALE was identified by sequence homology with human GALE (HGALE) while subsequent characterization revealed the enzyme to be 371 amino acids long, with a molecular weight of 40.6 kDa. As was observed for the previously mentioned AnUGM deletion strain, single GALEΔ and double (UGMΔ and GALEΔ) *A. nidulans* knockout studies both seriously perturbed cell growth and sporulation, which significantly reduced the overall fitness of the strains [34].

In order to identify potentially exploitable differences for drug targeting purposes, detailed structural characterization of *A. nidulans* GALE is required. To this end, we have determined the ternary crystal structure of AnGALE complexed with NAD^+^ and UDP-Glc*p*. The homodimeric structure is similar to the Human enzyme and mutational kinetic analysis of key residues responsible for anchoring co-factor (K160V) and serving as the active site base (Y156F) indicate a similar catalytic mechanism established for HGALE is likely operational within AnGALE. We also show that a ‘gate-keeper’ residue, namely L320 (*cf* C307 in HGALE and Y299 in *E. coli* GALE (EcGALE), can be mutated to accommodate interconversion of the larger UDP-N-acetylglucosamine/UDP-*N*-acetylgalactosamine (UDP-Glc*p*NAc/UDP-Gal*p*NAc) substrate pair. Kinetic values for UDP-Gal*p* turnover and inhibition by UDP-Gal*p*NAc and UDP-Glc*p*Nac are reported for wild-type and mutant AnGALE. Lastly, docking studies have been conducted on both UDP-Glc*p*/UDP-Gal*p* and UDP-Glc*p*NAc/UDP-Gal*p*NAc substrate pairs within the AnGALE structure and both L320C and L320Y modelled mutants which both support the overall activity profile and reinforce the inhibition results.

## Materials and Methods

### Protein Expression and Purification

Plasmid construction and DNA transformation of AnGALE was conducted according to previously published methods [34]. Overexpression of the N-terminal p-HIS-TEV-GALE was carried out in BL21-Gold (Novagen) cells and purified as previously described [33]. The HIS-tags employed for protein purification were not cleaved prior to crystallization trials. In short, the recombinant protein, as a 1L LB culture with kanamycin, was overexpressed at 15°C by induction with 0.2 mM IPTG and grown for 24 h at 250 rpm. The cells were collected and then lysed by sonication before removal of the cell debris by centrifugation. The resulting supernatant was filtered prior to loading onto a Protino Ni-IDA hand-packed column (Macherry-Nagel) at room temperature. The protein was eluted as one large single peak by imidazole gradient (0-250 mM) at a flow rate of 5 mL/m. The protein was analyzed by SDS-PAGE and the purest fractions pooled before being dialyzed against 25 mM Tris pH 8. The protein was concentrated with Vivaspin 20 centrifugal filter (30 kDa MWCO PES, Sartorius Stedim) to a final concentration, as determined by Bradford assay, of 15 mg/mL before being flash frozen in liquid N_2_.

### Mutant Production and Purification

Site-directed mutagenesis of AnGALE (Y156F, K160V, L320Y, and L320C) was performed using the QuikChange™ Site-Directed Mutagenesis Kit (Stratagene) according to the manufacturer’s protocol. Primers used to generate the alleles were designed as per the procedure outlined by the manufacturer and are as follows: Y156F-forward: 5' GGCCCGACAAACCCCTTCGGAAACACCAAGTTC 3', Y156F-reverse: 5' GAACTTGGTGTTTCCGAAGGGGTTTGTCGGGCC 3'; K160V-forward 5' CCCCTACGGAAA-CACCGTCTTCGCCATTGAGCTGG 3', K160V-reverse: 5' CCAGCTCAATGGCGAAGACGGTGTTTCCGTAGGGG 3'; L320Y-forward: 5' CCGGTGACGTCCTTAACTACACTTCTAACCCGACCCG 3', L320Y-reverse: 5' CGGGTC-GGGTTAGAAGTGTAGTTAAGGACGTCACCGG 3'; L320C-forward: 5' CCGGTGACGTCCTTAACTGCACTTCTA-ACCCGACCCG 3', L320C-reverse: 5' CGGGTCGGGTTAGAAGTGCAGTTAAGGACGTCACCGG 3'. The overexpression vector p-HIS-TEV-GALE was employed as the template DNA. PCR amplifications were carried out using 50 ng of the isolated template DNA and 15 pmol of each primer in a GeneAmp PCR PTC100 System (MJ Research, Inc.). The methylated DNA was digested with DpnI and subsequently 5 µL of each reaction were used for the transformation into *E. coli* DH5*α* competent cells (Novagen). Single colonies were selected from kanamycin resistant cultures grown on LB plates and the specific mutations were verified by DNA sequencing (NRC-PBI). For protein overexpression purposes, the isolated plasmid DNA was further transformed into BL21-Gold cells (Novagen). The mutants were purified in an identical manner to that outlined for the wild-type enzyme above [33].

### Crystallization and Cryoprotection

Broad screening trials were conducted with the use of commercial crystallization kits (Qiagen) via microbatch method at 277 K. Full details on the successful range of crystallization hits and experimental conditions have been published previously [33]. In brief, hexagonal rod-shaped crystals were obtained from crystallization drops consisting of equal volumes of protein and precipitant solution (20% (w/v) PEG3350, 0.1 M Bis-Tris propane pH 7.5, 0.2 M sodium fluoride) which had been layered with paraffin oil (Hampton Research). The crystals grew to dimensions of 0.1 x 0.1 x 0.4 mm over a period of two weeks. Optimized grid screens, based on the above condition, were also setup in the presence of substrates, specifically 10 mM UDP-galactose or UDP-glucose, which had been incubated with the enzyme for 1 h prior to setup. Although a variety of potential cryoprotectants were screened, the most suitable, as judged by the quality of the resulting X-ray diffraction pattern, was found to be 25% glycerol. Samples were quickly transferred into mother-liquor containing the cryoprotectant and after a few seconds were mounted onto a CryoLoop (Hampton Research) which was then flash-cooled in liquid N_2_.

### Data Collection and Processing

Data collection was carried out at 100 K at the Canadian Light Source (CLS) 08ID-1 beamline equipped with a MAR Mosaic MX-300 CCD detector. A 2.8 Å resolution data set for wild-type AnGALE was collected from 360 images with an exposure of 1 s and 1.0° oscillation at a crystal-to-detector distance of 250 mm. Examination of the diffraction patterns from multiple data sets revealed the consistent presence of nonmerohedral twinning due to the presence of a weaker second lattice. Ultimately, d*TREK was used to process the stronger lattice data by employing a cutoff rejecting data from integration and scaling which was less than eight times the standard deviation [35]. Relevant data collection statistics from one of the AnGALE crystals grown in the presence of UDP-Glc*p* are presented in Table 1. The slightly elevated R_merge_ could be due to residual twinned data that was not completely accounted for during data processing. Full data sets were also collected for crystals grown in the presence of UDP-Gal*p* and in the absence of substrate, however these data sets were of significantly lower resolution.

**Table 1:** Data collection and refinement statistics.

Beamline	08ID-1, CLS
Wavelength (Å)	0.9790
Temperature (K)	100
Space Group	C2
Unit-cell parameters (Å, °)	*a*=66.13, *b*=119.15, *c*=161.42, β=98.48
Resolution (Å)	36.71-2.80 (2.90-2.80)
Observed reflections	213151
Unique reflections	30056
Completeness (%)	98.4 (98.5)
Multiplicity	7.09 (7.34)
Mean *I*/*σ*(*I*)	8.1 (3.2)
*R*_merge_ †	0.131 (0.477)
*R*_meas_ ‡	0.142 (0.514)
Monomers per ASU	3
Resolution range (Å)	36.71-2.80
*R*_work_/*R*_free_ %	21.3/26.2
Number of protein residues	2-365, 3-365, 2-365
Number of solvent molecules	74
Ligands	3*NAD, 2*UDP/1*UDP-Glc*p*, 9*I, 1*GOL
*R*_msd_ bond length (Å)/angles (°)	0.002/0.560
Ramachandran favoured (%)	94.8
Ramachandran allowed (%)	4.5

Values in parentheses are for the highest resolution shell.

† *R*_merge_ = Ʃ_*hkl*_Ʃ_*i*_|*I*_*i*_(*hkl*) - <*I*(*hkl*)>|/Ʃ_*hkl*_Ʃ_*i*_ *I*_*i*_(*hkl*), where *I*_*i*_(*hkl*) is the measured intensity and <*I*(*hkl*)> is the average intensity over symmetry-equivalent reflections.

‡ *R*_meas_ = Ʃ_*hkl*_(√(*n*_*h*_/(*n*_*h*_ - 1)) Ʃ_*i*_|*I*_*i*_(*hkl*) - <*I*(*hkl*)>|/Ʃ_*hkl*_Ʃ_*i*_ *I*_*i*_(*hkl*)

### Structure Determination and Refinement

**Figure 2:**
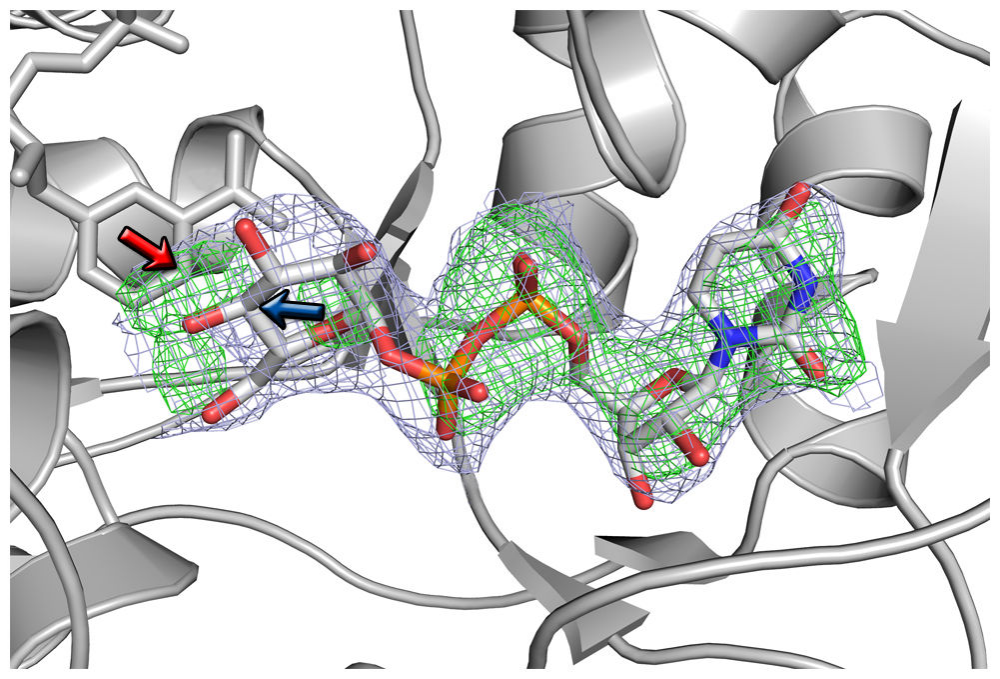
Electron density maps for UDP-Glc*p* modelled into the active site. The σ_A_-weighted 2|F _o_|-|F _c_| map (light purple) is contoured at 1σ while the σ_A_-weighted |F _o_|-|F _c_| map (green) is contoured at 3σ. The C4 position of NAD^+^ has been denoted with a red arrow while the sugar C4 is marked with a blue arrow.

The structure was solved by molecular replacement using *MrBUMP* [36] within the CCP4 package [37]. The initial solution was determined via MOLREP [38] employing human GALE (PDB entry 1HZJ) [39], with which AnGALE shares 51% sequence identity, as the template. The structure was refined through PHENIX [40] with initial rounds employing rigid body refinement followed by simulated annealing using Cartesian dynamics at 5000 K to remove model bias. At this point, clear positive density was observed in the electron difference maps contoured at 3σ for both NAD^+^ and bound substrate (UDP-sugar). Initial restrained refinement was conducted and then NCS restraints were applied throughout the remainder of the refinement process. Rebuilding and model manipulation were carried out in COOT [41]. Placement of cofactor and substrate within the model was conducted via *ligandFit* [42] and examined in COOT. Initially, UDP-Glc*p* was modeled into each of the three molecules in the ASU. Subsequent rounds of refinement revealed negative density in the difference maps for the substrate sugar moiety in two out of three monomers. As such, two of the monomers (chains B and C) were fitted with UDP while the third contains UDP-Glc*p*. Omit maps for the substrate region (Figure 2) were generated and examined to confirm the best assignments had been made given the quality of the data. An attempt to model UDP-Gal*p* into the substrate density was also made, however the fit was poor and negative density in the difference map appeared upon refinement. The libraries for NAD^+^, UDP-Glc*p*, and crystallized glycerol (GOL) were generated from ELBOW [43] in PHENIX [40]. Refinement progress was evaluated by following *R*_free_ with visual inspection of the electron density maps. Unmodelled electron density was examined with COOT which led to the placement of nine iodide ions (I¯) and one glycerol moiety within the ASU. The iodide source was likely a contaminant from the sodium fluoride employed in the crystallization condition. Water molecules were included in the refinement via update waters in PHENIX once *R*_free_ dropped below 30%. The water positions were subsequently evaluated by manual inspection through COOT. Optimized refinement target weights for best geometry were employed in the final round of refinement. Stereochemical validation of the structural model was conducted with MOLPROBITY [44] within PHENIX. Final refinement statistics are given in Table 1. All of the images were generated with PyMOL [45]. The atomic coordinates and structure factors for the AnGALE model have been deposited within the Protein Data Bank with accession code: 4LIS.

### HPLC and Kinetic Activity Assay

**Figure 3:**
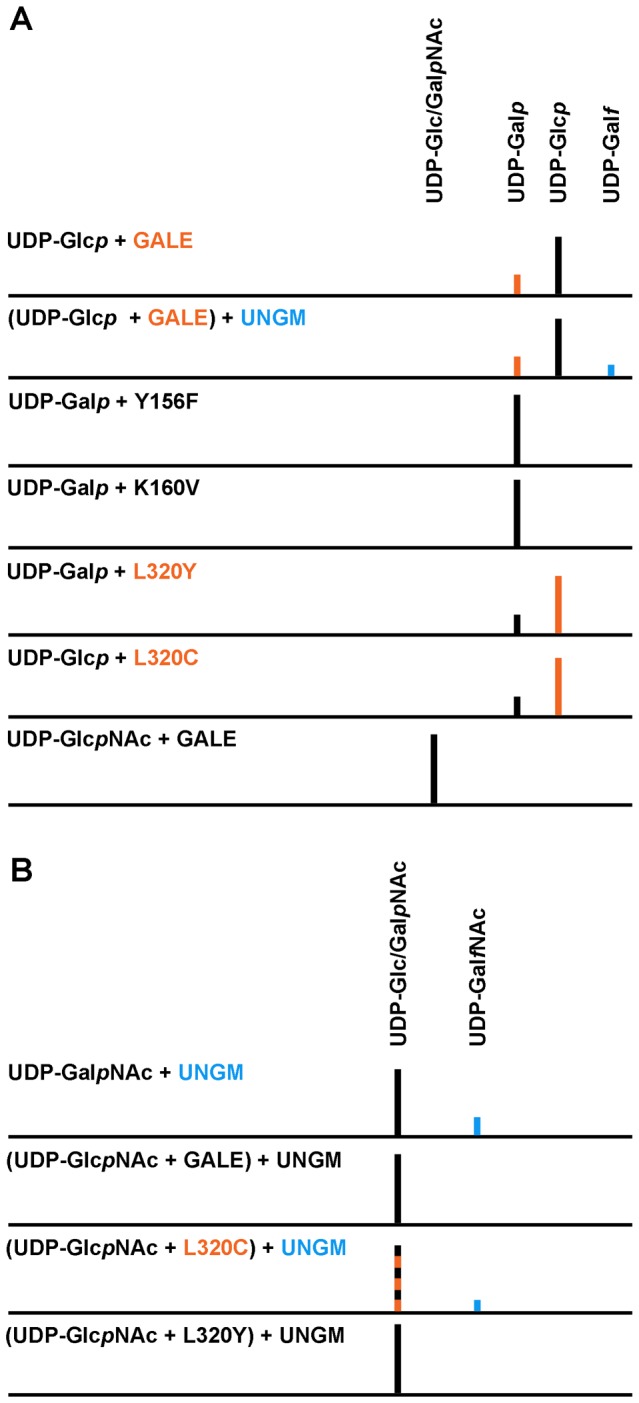
Schematic of the initial HPLC studies for AnGALE. a) Examination of UDP-Glc*p*/UDP-Gal*p* substrate interconversion for wild-type and mutant enzymes. b) UNGM coupled reaction for monitoring interconversion of the UDP-Glc*p*NAc/UDP-Gal*p*NAc substrate pair. Colors represent both product formation and the enzyme responsible for the catalytic reaction.

The UDP-Glc*p*/UDP-Gal*p* interconversion was monitored by HPLC (Waters) using a CarboPac PA1 (Dionex Inc.) column with either 150 mM ammonium acetate buffer (pH 8) or 200 mM ammonium acetate buffer (pH 7). Generally, the former buffer was employed for better separation of the pyranose-based compounds (UDP-Gal*p*: 51.1 and UDP-Glc*p*: 56.1 minutes) while the later buffer was ideal for separating the UNGM coupled reaction product (UDP-Gal*p*: 13.3, UDP-Glc*p*: 14.5, and UDP-Gal*f*: 18.0 minutes) described next. HPLC investigation of the UDP-Glc*p*NAc/UDP-Gal*p*NAc interconversion was performed on a Gemini 5u C18 (Phenomenex USA) column with 50 mM triethylammonium acetate buffer (pH 6.5) containing 1.5% acetonitrile. UDP-N-acetylgalactopyranose mutase (UNGM) was coupled to the interconversion reaction (UDP-Glc*p*NAc: 10.9 and UDP-GalpNAc: 11.0 minutes) so that a clearly separable product could be measured (UDP-Gal*f*NAc: 13.3 minutes). Chromatographic runs were performed at 25°C, unless otherwise stated, with a mobile phase flow rate of 1 mL/m. Individual standards were run in each of the buffer systems to ensure correct peak assignments had been made. The chromatographic data, which has been normalized on account of the different buffer systems and columns employed in the study, is presented schematically in Figure 3 for ease of viewing.

Kinetic studies were performed as outlined previously [46] with slight changes to the method and modifications for use in single continuous sampling mode on a Cary 50 Bio (Varian) UV-Vis spectrometer. Wild-type AnGALE and mutant activity were assayed by incubating a mixture consisting of NAD^+^ (2 mM, Calbiochem), UDP-Glucose dehydrogenase (250 µU/µL, Calbiochem), Tris-HCl (100 mM, pH 8.5, Aldrich), while varying UDP-Gal*p* (50 µM to 1 mM, Calbiochem) for 10 minutes at 25°C before AnGALE (2 µg/mL) was added to initiate the reaction. The incubation period assured the conversion of any potentially contaminating UDP-Glc*p* from the assay. Spectrosil® Quartz MicroCells (Starna) were employed for the assays with a total reaction volume of 500 µL. As the irreversible oxidation of UDP-Glc*p* to UDP-glucuronic acid results in the reduction of two NAD^+^ molecules per molecule of UDP-Glc*p*, the reaction was followed spectrophotometrically at 340nm. Assays were measured over a 5 minute period while the rates (v) were calculated by fitting the linear portion of the *A*_340_ vs. time plot, post initial lag phase, to a straight line after correction for baseline absorption. Saturating conditions were verified by repeated doubling of both the substrate and coupled enzyme while assay linearity in the range used was confirmed with different dilutions of AnGALE. After unit conversion, the apparent kinetic constants *K*_m_ and *k*_cat_ were derived by Michaelis-Menten nonlinear curve fitting with Prism 3.0 Software (GraphPad, San Diego CA, USA). Inhibition studies of UDP-Gal*p* conversion within wild-type and mutant AnGALE by UDP-Glc*p*NAc and UDP-Gal*p*NAc were performed according to previously established methods [47]. In short, 0.5mM of either UDP-Glc*p*NAc or UDP-Gal*p*NAc was added to each experimental assay described above to act as a competing substrate. Rates at the various UDP-Gal*p* concentrations were determined and the apparent kinetic constants were calculated as described previously and employed in the calculation of *K*_i_. Studies have shown that determination of *K*_i_ for a given competitive substrate in this manner gives a close approximation to its apparent *K*_m_ [48,49].

### Docking Studies

Binding mode predictions for both UDP-Glc*p*/UDP-Gal*p* and UDP-Glc*p*NAc/UDP-Gal*p*NAc substrate pairs within wild-type and mutant modelled AnGALE along with HGALE and EcGALE were examined with the aid of GOLD Suite (Cambridge Crystallographic Data Centre) [50]. Validity of the docking experiments was established through reproduction of observed binding modes within previously reported crystal structures of both human (UDP-Glc*p*: 1EK6, UDP-Glc*p*NAc: 1HZJ) and *E. coli* (UDP-Glc*p*: 1XEL, UDP-Glc*p*NAc: 1LRJ) GALE-ligand complexes along with the AnGALE structure. In all cases, protein models were first protonated and then examined to ensure docking site residues had been assigned correct ionisation and tautomeric states. Given the lack of binding site hydration, water molecules were removed from the models to facilitate the docking process. Ligand molecules occupying the docking site of interest were extracted and reloaded separately for the purpose of defining the binding site as any residues having atoms within 6Å of the ligand. In an effort to optimize the docking, as the L320Y and L320C protein models were generated by simple mutations within COOT, these residues were specified as ‘flexible side chains’ with constraints from the predefined GOLD rotamer library [51]. Additionally, residue N219 was also set to flexible with library constraints when docking the larger *N*-acetylated substrate pair. Atomic parameters for the ligands employed in the docking studies were extracted from previous crystal structures. After ensuring both atom and bond types were correct, hydrogen atoms were added and the ligands examined for inconsistencies. The CHEMPLP Fitness Function was chosen as it reproduced observed binding modes most reliably during validity testing. Default values for both ligand search options and flexibility were employed while constraints were not used in the docking. In order to ensure high predictive accuracy the ligand-dependent genetic algorithm search efficiency was set to 200% (very flexible). In all cases, the predicted binding modes reported herein were from the top solutions as defined by the overall fitness score.

## Results and Discussion

### Overall Structure

**Figure 4:**
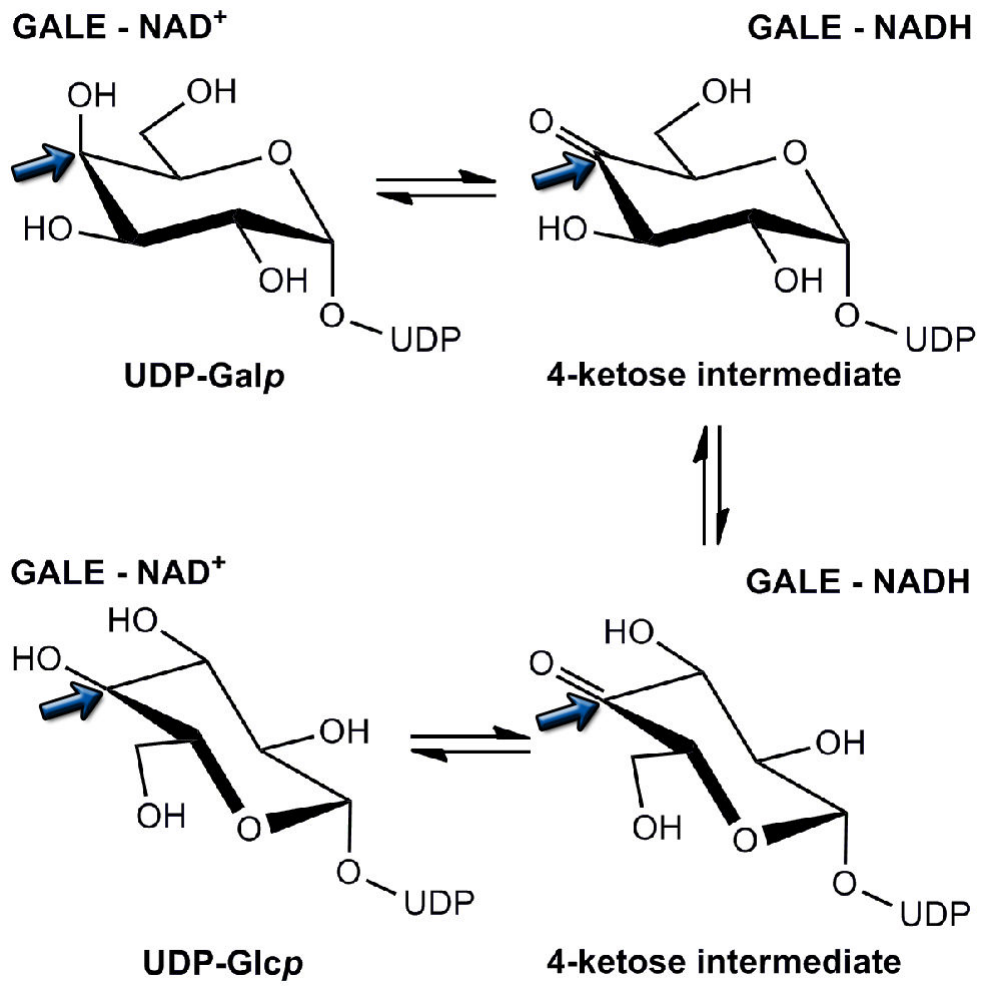
Substrate interconversion within GALE occurs via a well-established three step process. The C4 position of the sugar in each molecule is highlighted by a blue arrow.

The majority of GALE crystallographic studies have focused on the homodimeric structure initially identified within the *E. coli* species (EcGALE) and the more recently examined Human form (HGALE). However, a limited number of structures have appeared in the literature from additional species, including *Pseudomonas aeruginosa* [52], *Trypanosoma brucei* [53,54], *Saccharomyces cerevisiae* [55], and more recently *Pyrobaculum calidifontis* [56]. The initial structural studies revealed GALE requires tightly, non-covalently bound nicotinamide adenine dinucleotide (NAD^+^) co-factor for catalytic activity and also established the enzyme as part of the short chain dehydrogenase/reductase (SDR) superfamily [57-59]. Such proteins are characterized by a conserved Y-X-X-X-K catalytic motif and a signature G-X-X-X-G-X-G motif for co-factor binding [60,61]. In depth structural and biochemical studies suggest a three-step mechanism for the catalytic conversion (Figure 4) which involves: 1) hydrogen abstraction of the 4'-hydroxyl group by tyrosine of the conserved Y-X-X-X-K motif and hydride transfer from C4 of the sugar to C4 of NAD^+^; 2) rotation of the 4-ketose intermediate by 180° within the active site positions the opposite face of the sugar moiety toward the reduced dinucleotide; and 3) hydride transfer from the nicotinamide ring of NADH back to C4 of the sugar and reprotonation of the 4'-hydroxyl by tyrosine [62-64].

**Figure 5:**
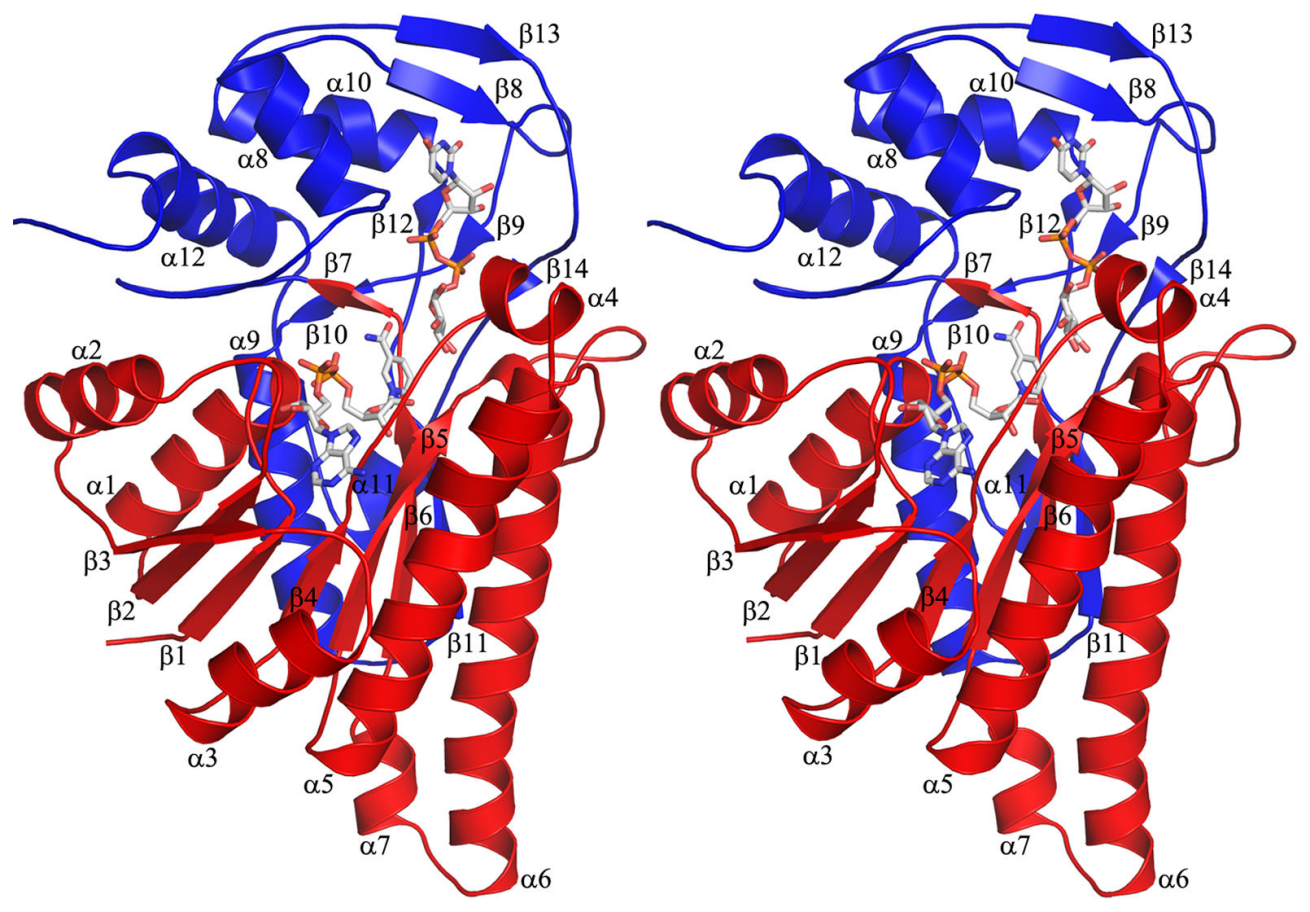
Stereoscopic ribbon depiction of a representative AnGALE monomeric unit. Each subunit of the dimeric enzyme contains an N-terminal (red) and C-terminal (blue) domain. The co-factor and substrate appear in stick representations.

AnGALE was found to crystallize in the C2 space group with an asymmetric unit containing the characteristic homodimer previously reported in both the *E. coli* and Human forms, along with an additional monomer which also forms a dimer through symmetry. A ribbon representation of the AnGALE monomer containing UDP-Glc*p* is depicted in Figure 5. The three monomers are nearly identical with backbone root-mean-square deviation (r.m.s.d.) of 0.2, 0.4, and 0.2 Å between monomer A & B, monomer A & C, and monomer B & C, respectively. As can be seen, the structure folds into two domains consisting of an N-terminal motif (M1 - A201, red) and C-terminal motif (G202 - K371, blue). While the dimer interface is formed between pairs of the longest α-helices (α5 and α6) within adjacent N-terminal domains, the domain itself consists of seven α-helices (6-26 residues in length) and seven β-strands (3-7 residues in length). The main feature of the N-terminal domain is a modified Rossmann fold constructed from a centralized seven-stranded parallel β-sheet (β3, β2, β1, β4, β5, β6, and β11) flanked by two α-helices (α1 and α2) on one side and three on the other (α3, α5, and α6). In addition, there is a short α-helix (α4), a 3/10-helix (η1), and a mixed domain parallel β-sheet (β7 and β10) above the main β-sheet, while below resides another short α-helix (α7). The NAD^+^ is anchored within this domain directly atop the β-sheet forming one half of the active site which exists in the cleft between the domains. The C-terminal domain positions the substrate in the correct relative orientation for catalysis within the cleft and consists of five α-helices (5-17 residues in length) and a total of seven β-strands (2-6 residues in length). The β-strands in this domain are arranged into a two-stranded parallel β-sheet (β8 and β13) and a short three-stranded β-sheet (β12 antiparallel, β9 and β14 parallel) along with the mixed domain parallel β-sheet (β10 and β7). The seventh strand of the β-sheet defining the N-terminal Rossmann fold comes from the C-terminal domain (β11).

**Figure 6:**
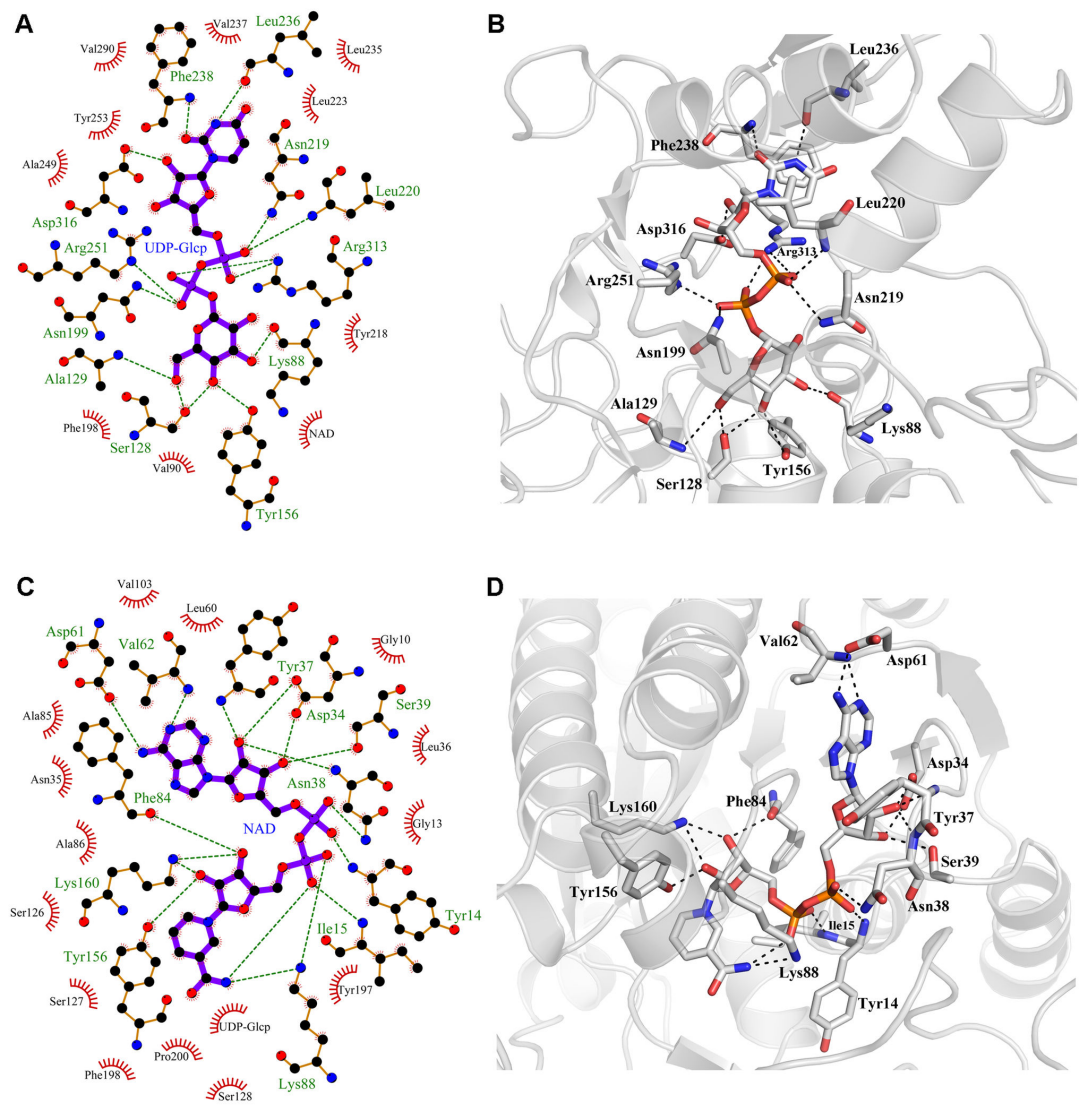
Interactions anchoring the cofactor and substrate within the active site of AnGALE. (**a**) **Schematic and (b) model representations of the UDP-Glc*p* binding**. (**c**) **Schematic and (d) model representation of the NAD^+^ binding**. For the schematic representations hydrogen bonds are shown in green and hydrophobic interactions in red. Hydrogen bonds are depicted as dashed lines in the model representations.

A schematic of the immediate environment about the NAD^+^ binding site is depicted in Figure 6. There are a total of 16 hydrogen bonds anchoring the cofactor within the N-terminal portion of the active site cleft. More specifically, seven side chains (D34, N38, S39, D61, K88, Y156, and K160), five backbone NH groups (Y14, I15, Y37, N38, and V62), and one backbone CO group (F84) participate in the nucleotide hydrogen bonding. In addition, there is one intramolecular bond between the amide nitrogen of the nicotinamide group and an oxygen of the β-phosphate. Two water molecules also interact directly with the NAD^+^, one forms hydrogen bonds to both phosphates while the second contacts the nicotinamide moiety. Inspection of the model revealed the nicotinamide group of the cofactor is in the *syn*-conformation while both ribose units adopt a C_2'_-endo conformation. It should be noted that the two main contributors with disallowed Ramachandran dihedrals, namely Y14 and F198, reside in the region surrounding the cofactor.

The substrate binding environment is shown schematically in Figure 6 where a total of 14 hydrogen bonds anchor the UDP-Glc*p* within the C-terminal portion of the active site cleft. While the uracil moiety is held in place through π-π stacking (F238) and hydrogen bonding contributions from backbone NH (F238) and CO (L236) groups, the 2'-hydroxyl of the ribose forms a contact with the carboxylate of D316. The phosphate region participates in five hydrogen bonding interactions with four nearest side chains (N199, N219, R251, and R313) and one backbone NH (L220). The sugar portion of the substrate is within hydrogen bonding distance of four residues. Hydroxyl groups O3' and O6' both interact with the protein backbone, namely CO of L88 and NH of A129 respectively, while S128 forms a bifurcated contact (O4' and O6') and Y156 serves as the active base for O4'

### Structural Comparison

**Figure 7:**
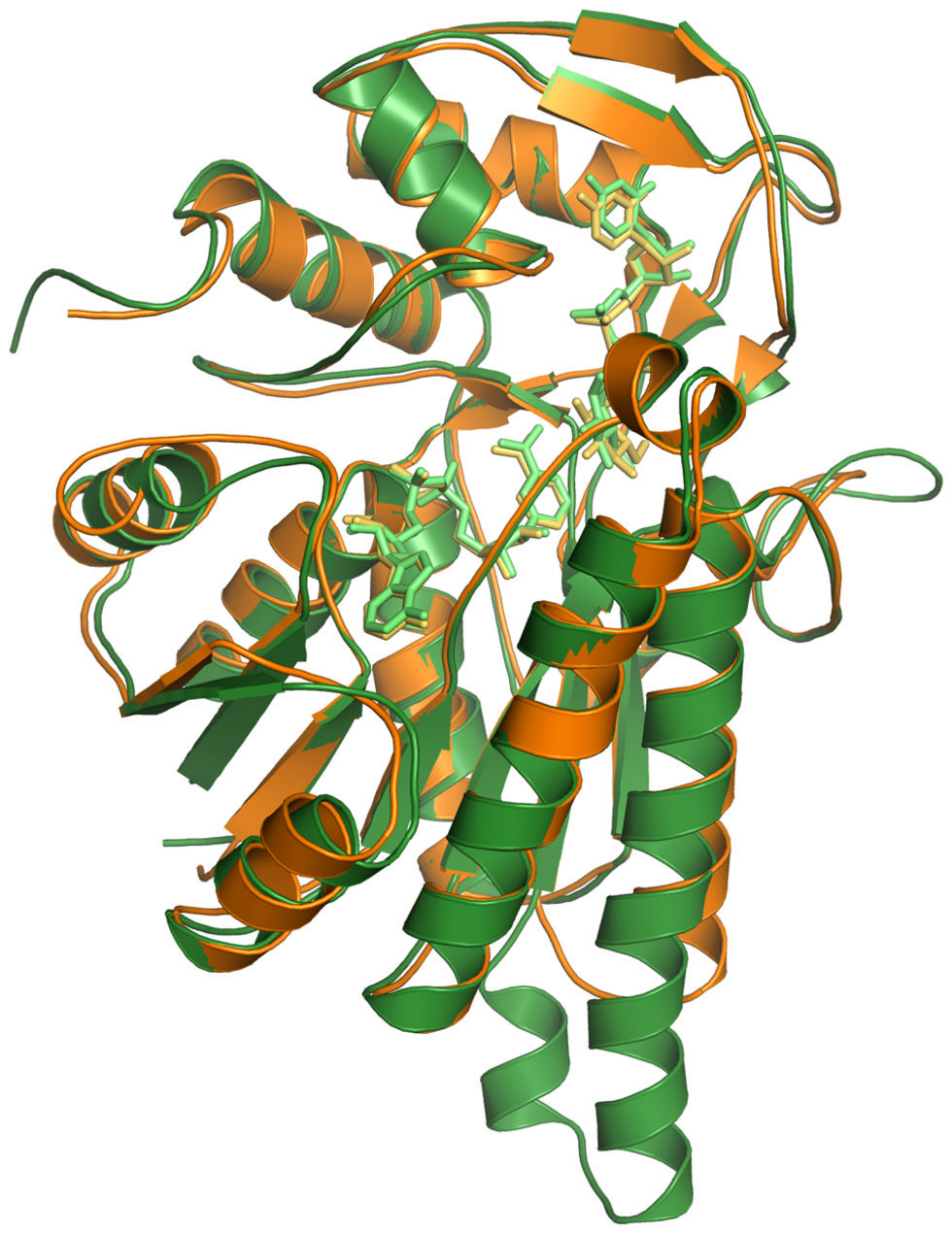
Superposition of the monomeric ribbon representation for AnGALE (green) and HGALE (orange). Cofactor and substrates for both enzymes are depicted in lighter variants as stick representations for clarity.

**Figure 8:**
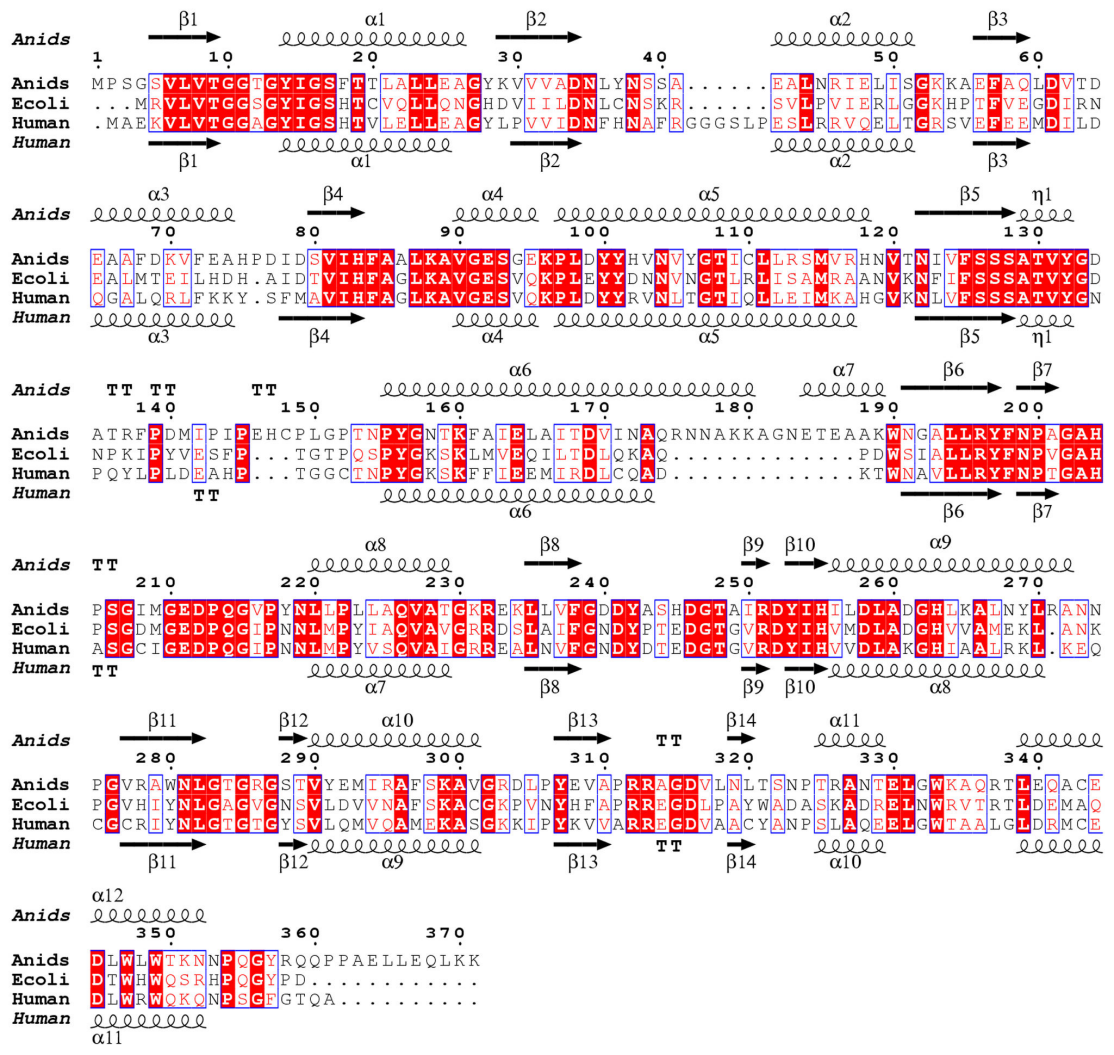
Structure-based sequence alignment of *A. nidulans*, *E. coli*, and Human forms of GALE. Identical residues are highlighted in red, similar residues are red surrounded with blue boxes.

In order to gain insight into the structure-function relationship operating in the fungal enzyme, the ternary AnGALE structure was compared to Human GALE (HGALE), for which mechanistic and structural studies have been well established. The HGALE ternary complex (PDB code 1EK6) with bound UDP-Glc*p*, was employed for comparison. A superposition of the monomeric ribbon representation for AnGALE and HGALE is displayed in Figure 7. For clarity, the structure-based sequence alignment is presented in Figure 8 which also includes the well-studied ternary EcGALE structure (PDB code 1XEL) for completeness. Examination of the structures reveals the models are remarkably similar, the major difference being that AnGALE has an elongated and slightly bent α6 helix along with an additional short α7 helix prior to β6 compared to HGALE. Also, AnGALE has an elongated loop in the region delineated by A135 - P143 (cf. P139 - P143 in HGALE) and is missing a short loop prior to the α2 helix (G41 - P46 in HGALE).

Superpositioning of the two crystallographically unique HGALE chains with the AnGALE UDP-Glc*p* monomer resulted in a r.m.s.d. of 1.1 and 1.3 Å over 339 equivalent C-α positions. The EcGALE structure was also remarkably similar to AnGALE having an r.m.s.d. of 1.1 Å over 337 equivalent C-α(s). Previous structural studies have shown the difference in r.m.s.d. between the two HGALE chains is due to the C-domain of one monomer clamping down more tightly over the active site. The end result placed the postulated active site base closer to the 4'-hydroxyl group of the substrate which ultimately helped to establish the currently accepted catalytic mechanism. Although the AnGALE backbone exhibits a closer match with the less tightly closed HGALE monomer (1.1 Å r.m.s.d.), the active site interactions of the more tightly closed monomer, which helped establish the HGALE catalytic mechanism, were also observed in AnGALE. The backbone deviations between the HGALE chains and the AnGALE monomer are limited primarily to the C-terminal regions forming direct contacts with the substrate and are defined by G210 - A243 (including α8 and β8) and V301 - L318 (including β13).

Given the N-terminal structural similarity shared between AnGALE and HGALE, it is not surprising that the cofactor binding is remarkably similar in both enzymes. In particular, the NAD^+^ conformations of the nicotinamide ring (*syn*) and both ribose units (C_2'_-endo) were previously observed within the structures of the Human and *E. coli* counterparts, both of which are known to be *B*-side specific enzymes. In comparison to HGALE, the AnGALE model features two additional cofactor anchoring points in Y37 and S39, both of which form contacts with ribose OH groups, along with an additional interaction between K88 and the nicotinamide functionality. The remaining residues are identical in composition and form equivalent hydrogen bonding patterns, the only exception being that V62 is replaced by I67 in HGALE. Examination of the cofactor binding pockets revealed AnGALE, with a total of 16 hydrogen bonding interactions, to represent the median between HGALE and EcGALE which have 13 and 18 contacts, respectively.

The more tightly closed HGALE monomer was employed for substrate binding comparison due to the closer binding similarity with AnGALE. Although the number of residues involved in substrate binding is comparable, both the composition and hydrogen bonding patterns are slightly different in the two enzymes. Overall, 14 interactions anchor the substrate within AnGALE compared to 17 hydrogen bonds observed within HGALE. While the only difference within the nucleoside binding region is the replacement of the L236 backbone CO contact with a similar N224 interaction within HGALE, the phosphate region of HGALE participates in an additional interaction with the second NH_2_ group of R300 (R313 in AnGALE). In the glucosyl region, the HGALE substrate 2'-hydroxyl group forms two hydrogen bonds, one with the side-chain of N207 (equivalent is N219) and one with the backbone CO of K92 (equivalent to K88) which are not observed in the AnGALE structure. In addition, the HGALE active site base Y157 (equivalent is Y156) also forms a contact with the 3'-hydroxyl of the substrate which is not observed in AnGALE. Lastly, while the AnGALE substrate 6'-hydroxyl interaction with S128 (equivalent is S132) is not seen in HGALE, the backbone NH interaction of A129 is replaced in HGALE with a second contact from the side-chain of N187 (N199 in AnGALE).

Evidence supporting AnGALE operating in a similar fashion to that previously established for HGALE comes from examining the interactions directly associated with key catalytic steps in the mechanism. Focusing on the reaction center, Y156, postulated to be the active site base in the Human enzyme (Y157), forms a similar hydrogen bond to the substrate 4'-hydroxyl group (3.4 Å) previously observed in HGALE (3.2 Å). Also, the S128 contact (2.8 Å), which was reasoned to facilitate proton transfer between the 4'-hydroxyl and the active site base, was also found in HGALE (S132, 2.5 Å). Furthermore, the equivalent hydrogen bond between Y157 and the NAD^+^ nicotinamide nitrogen (3.7 Å) in HGALE, which was believed to lower the pK_a_ allowing it to function directly as the active site base, was also found in AnGALE (Y156, 3.7 Å). Also of importance to the reaction mechanism is the hydride transfer distance between C4 of the sugar and C4 of the nicotinamide ring which was found to be 3.5 Å in both enzymes.

### Kinetic Assessment of AnGALE

Epimerase activity for both the wild-type and mutant enzymes was initially investigated by HPLC before being assayed spectrophotometrically. A schematic of the HPLC results is presented in Figure 3. As outlined, AnGALE was able to interconvert between UDP-Glc*p* and UDP-Gal*p in vitro* resulting in a 75:25 (UDP-Glc*p*:UDP-Gal*p*) mixture regardless of which substrate was employed (reverse reaction not shown). In an effort to establish mechanistic similarity between AnGALE and HGALE, mutants of the conserved Y-X-X-X-K catalytic motif, namely Y156F and K160V, were tested for activity. As was established for previous epimerases, both mutants were unable to perform the interconversion and as such the associated residues are deemed intrinsically linked to the catalytic mechanism.

Previous studies have shown GALE to exhibit inter-species variation with respect to size and shape selectivity of potential substrates. While both EcGALE and HGALE are capable of interconverting UDP-Glc*p*/UDP-Gal*p* with equal efficiency, HGALE can also interconvert UDP-Glc*p*NAc/UDP-Gal*p*NAc [48,65,66]. Structurally, it was shown that N207 of the Human enzyme shifts to accommodate the N-acetyl group and that the active site cleft volume is 15% larger in HGALE primarily due to a single amino acid difference, i.e. C307 in HGALE vs. Y299 in EcGALE [39]. The additional catalytic activity was attributed to the larger HGALE active site being able to accommodate entry and rotation of the N-acetylated substrate pair. The UDP-Glc*p*NAc/UDP-Gal*p*NAc interconversion selectivity for the two enzymes could be reversed through a point mutation to a single 'gatekeeper' residue. Kinetic measurements on the pertinent EcGALE Y299C mutant showed a >230-fold increase in activity for UDP-Glc*p*NAc/UDP-Gal*p*NAc interconversion with a concurrent 5-fold loss of activity for UDP-Glc*p*/UDP-Gal*p* [67]. Similarly, kinetic measurements on the relevant HGALE C307Y mutant revealed a complete loss of interconversion activity for the larger substrate pair while the smaller substrate pair demonstrated full wild-type activity [68]. It was noted for these systems, that while a small cleft size may preclude access, and hence activity towards larger substrates, the opposite is not necessarily observed.

In this regard, L320, the equivalent gatekeeper residue in AnGALE was mutated to mimic both EcGALE (L320Y) and HGALE (L320C) so that a comparative substrate selectivity profile could be established. Based on previous results, it was anticipated both mutants would interconvert UDP-Glc*p*/UDP-Gal*p* while the L320C mutant enzyme would also exhibit UDP-Glc*p*NAc/UDP-Gal*p*NAc interconversion and the L320Y mutant enzyme would not. Indeed, examination of the HPLC data revealed that both mutants were capable of interconverting UDP-Glc*p*/UDP-Gal*p*. Unfortunately, interconversion for the UDP-Glc*p*NAc/UDP-Gal*p*NAc pair by AnGALE was not separable by HPLC and as a result, the reaction was coupled with the enzyme UDP-N-acetylgalactopyranose mutase (UNGM) [69] to form a measurable product (UDP-Gal*f*NAc). UNGM functions in a similar manner to UGM catalyzing the production of UDP-Gal*f*NAc from precursor UDP-Gal*p*NAc. As is evident from Figure 3b, UDP-Gal*f*NAc was not observed for the coupled reaction with wild-type AnGALE, indicating the required interconversion for production of UDP-Gal*p*NAc precursor did not take place. The experiment was also performed at 37°C with the same results (not shown) to ensure the coupled reaction had reached equilibrium. Based purely on the relative size of the gatekeeper residues [70], the results of the wild-type coupled reaction for the larger substrate pair are not surprising, given that AnGALE (L320 volume =165Å^3^) more closely resembles EcGALE (Y299 volume =197 Å^3^) than HGALE (C307 volume =113 Å^3^). As was anticipated, the larger cleft L320C mutant, when coupled with UNGM, produced UDP-Gal*f*NAc while the equivalent smaller cleft L320Y coupled reaction was not active. Although the UDP-Glc*p*NAc/UDP-Gal*p*NAc interconversion could not be directly detected for L320C, UDP-Gal*f*NAc could only be produced when the converted UDP-Gal*p*NAc precursor is present in the reaction medium.

Based on the HPLC results, kinetic assays were performed on AnGALE as well as the mutants and the pertinent kinetic constants derived from the data are presented in Table 2. Previous GALE kinetic studies for UDP-Gal*p* from a variety of mammalian and microbial sources have reported *K*_m_ values ranging from 0.02 mM to 0.23 mM [47,62,71-73]. While the apparent *K*_m_ for wild-type AnGALE (0.11 ± 0.01 mM) was within the reported range, the *k*_cat_ (12.8 ± 0.6 s^-1^) was slower than that found previously for both HGALE and EcGALE. Ultimately, the catalytic efficiency of wild-type AnGALE was approximately 5 fold and 27 fold lower than HGALE and EcGALE, respectively. Interestingly, the L320Y mutant, which possesses a relatively smaller active site cavity compared to the wild-type enzyme, bound UDP-Gal*p* tighter (0.06 ± 0.01 mM) but the turnover rate was almost 9 times slower (1.5 ± 0.1 s^-1^). The tighter binding could be attributed to additional hydrogen bonding between the side chain of the mutated tyrosine and the C6-OH group of the substrate which was found to be active within the EcGALE structure (2.8 Å). The additional anchoring within AnGALE L320Y could hamper the key catalytic step, i.e. a 180° rotation of the 4-ketose intermediate, which may explain the slower rate and hence a more than 4 fold drop in catalytic efficiency.

**Table 2:** Apparent kinetic parameters for wild-type and mutant AnGALE with UDP-Gal*p* substrate.

	***K*_m_ (mM)**	***k*_*cat*_ (s^-1^)**	***k*_cat_/*K*_m_ (mM^-1^s^-1^)**
**AnGALE**	0.11 ± 0.01	12.8 ± 0.6	116.4 ± 8.1
**L320Y**	0.06 ± 0.01	1.5 ± 0.1	26.8 ± 1.9
**HGALE**	0.07 ± 0.01	36 ± 1	521.7 ± 72
**EcGALE**	0.16 ± 0.02	500 ± 50	3125 ± 312

Parameters for HGALE and EcGALE were taken from References [75] and [76], respectively.

**Figure 9:**
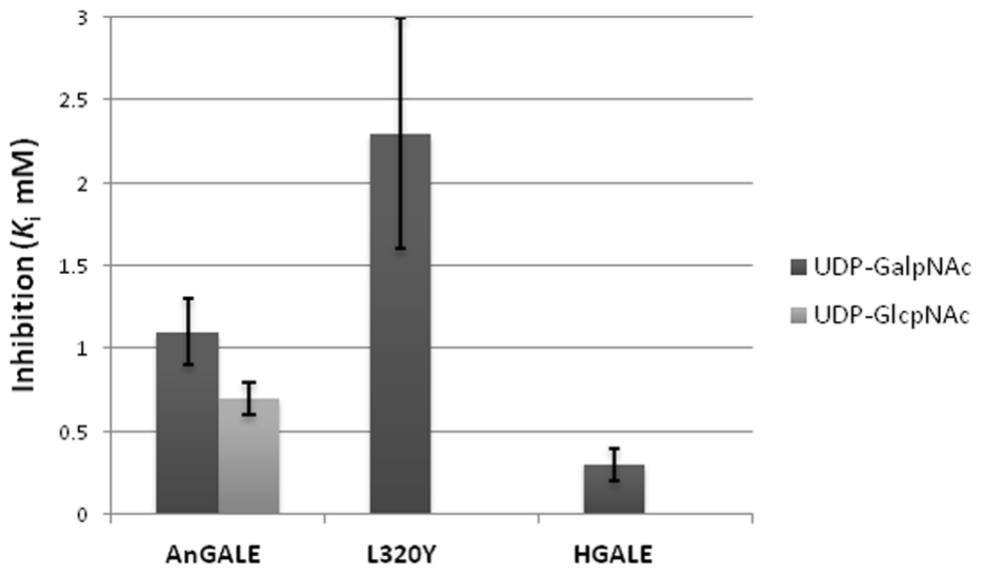
Competitive inhibition of GALE UDP-Gal*p* activity by UDP-Gal*p*NAc and UDP-Glc*p*NAc. The apparent *K*_i_ for HGALE was taken from Reference [47].

Inhibition of UDP-Glc*p*/UDP-Gal*p* interconversion for wild-type and L320Y mutant AnGALE by members of the larger UDP-Glc*p*NAc/UDP-Gal*p*NAc substrate pair was also examined and the results are presented in Figure 9. As can be seen, wild-type AnGALE inhibition is significantly weaker than that observed for wild-type HGALE. The stronger inhibition for HGALE is not surprising given the enzyme’s known ability to accommodate and interconvert members of the larger UDP-Glc*p*NAc/UDP-Gal*p*NAc substrate pair. The fact that competitive inhibition was observed for wild-type AnGALE reveals that, although interconversion was not observed, the larger substrates were able to weakly bind within the relatively smaller active site. In this context, the more than two fold decrease in inhibition within the L320Y mutant is not surprising, as there would be less room, and hence a smaller likelihood, for the inhibitors to bind efficiently. Unfortunately, due to discontinuation of the commercial source for the UDP-Glucose dehydrogenase enzyme, full kinetic and inhibition studies on the L320C mutant, which showed preliminary HPLC activity for both UDP-Gal*p* and UDP-Gal*p*NAc, could not be conducted.

### Probing Active Site Affinity

**Figure 10:**
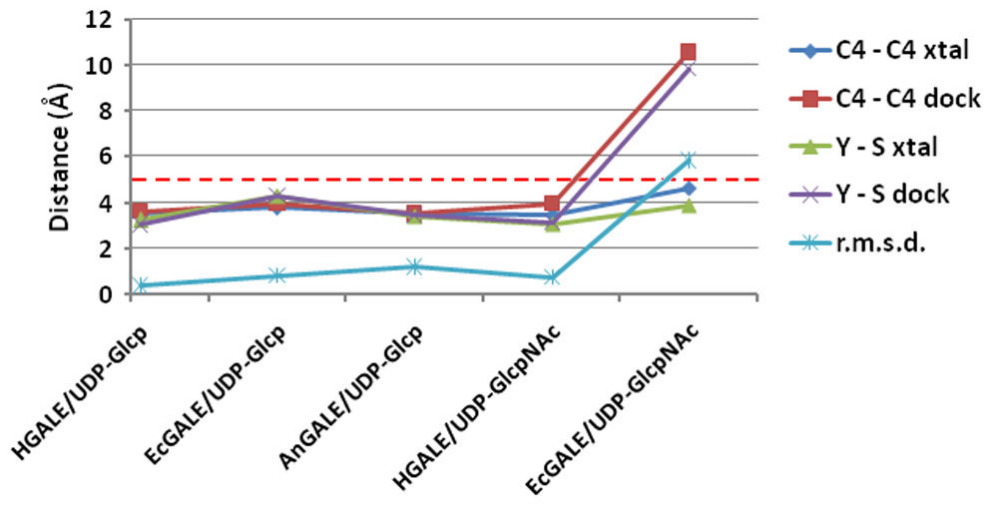
Validation of the docking procedure through reproduction of substrate binding modes. C4 - C4 refers to the distance between C4(s) of nicotinamide and sugar, while Y-S refers to the distance between OH(s) of Y156 and sugar C4. The dashed red line represents the upper limit criteria (5 Å) for evaluating productive poses. PDB codes for docking UDP-Glcp: HGALE (1EK6) and EcGALE (1XEL); UDP-GlcpNAc: HGALE (1HZJ) and EcGALE (1LRJ). All units are in Å.

Docking studies for the wild-type AnGALE structure and mutant models were conducted to establish potential binding modes for both pairs of substrates. In addition, binding mode predictions for HGALE and EcGALE were also performed for comparative purposes. Pertinent parameters extracted from the initial docking validation studies are presented in Figure 10. As can be seen, with the exception of the EcGALE/UDP-Glc*p*NAc docking discussed below, reproduction of observed binding modes was highly successful. As the UDP portion for each substrate docked in a near perfect manner, only the r.m.s.d. for the sugar moiety, which ranged from 0.3 to 1.1 Å, has been reported for evaluation. The exceedingly small deviations in sugar orientation translate into excellent agreement between predicted (dock) and observed (xtal) distances for both catalytically important contacts, namely the separation between C4(s) of the nicotinamide/sugar (d_C4-C4_) and the OH(s) of Y156/sugar C4 (d_Y-S_). The deviations in the EcGALE/UDP-Glc*p*NAc are not surprising, given that the substrate sugar moiety within the crystal structure had been modelled into disordered electron density. The lack of unambiguous sugar density could be attributed to the inability of the *E. coli* epimerase efficiently binding UDP-Glc*p*NAc in a productive mode, hence resulting in the enzyme’s inability to interconvert the larger N-acetylated substrate pair. Ultimately, the docking validation resulted in successful binding pose predictions for enzymes with established substrate activity and verification of non-productive binding modes for an inactive enzyme.

The comparative GALE docking results for both UDP-Glc*p*/UDP-Gal*p* and UDP-Glc*p*NAc/UDP-Gal*p*NAc substrate pairs are presented in Table 3. The catalytically important contacts, namely d_Y-S_ and d_C4-C4_, represent two of the criteria by which the binding modes were judged to be productive. The upper limit for both contacts to contribute towards a productive binding mode was deemed to be approximately 5 Å as this was roughly the maximum distance observed for these criteria in previous structures with GALE activity. The additional factor contributing to a productive binding mode was the relative orientation of the sugar moiety within the active site. Since the mechanism for the reversible reaction requires a 180° rotation of the sugar, it was anticipated that productive binding modes for members of each substrate pair would exhibit opposite, or flipped, sugar orientations. For convention, the relative sugar orientation observed within previously reported GALE UDP-Glcp structures is defined as having no flip. The predicted docking modes for both UDP-Glc*p* and UDP-Gal*p* for each enzyme examined showed the expected, productive binding mode. As such, the UDP-Glc*p*/UDP-Gal*p* docking results corroborate the experimentally determined activity profiles for GALE enzymes towards the UDP-Glc*p*/UDP-Gal*p* substrate pair.

**Table 3:** Docking results for both sets of substrate pairs within GALE structures.

**UDP-Glc*p***						**UDP-Gal*p***			
	**d_Y - S_ (Å)**	**d_C4 - C4_ (Å)**		**FLIP**	**Productive**	**d_Y - S_ (Å)**	**d_C4 - C4_ (Å)**	**FLIP**	**Productive**
**HGALE**	3.001	3.572		N	P	3.040	3.206	Y	P
**EcGALE**	4.271	3.921		N	P	3.860	3.403	Y	P
**AnGALE**	3.403	3.473		N	P	4.602	3.543	Y	P
**L320Y**	3.337	3.312		N	P	4.303	3.770	Y	P
**L320C**	4.424	3.881		N	P	4.482	3.793	Y	P
**UDP-Glc*p*NAc**						**UDP-Gal*p*NAc**			
	**d_Y - S_ (Å)**		**d_C4 - C4_ (Å)**	**FLIP**	**Productive**	**d_Y - S_ (Å)**	**d_C4 - C4_ (Å)**	**FLIP**	**Productive**
**HGALE**	3.097		3.922	N	P	4.698	6.218	Y	P
**EcGALE**	9.838		10.565	N	NP	10.726	10.804	Y	NP
**AnGALE**	7.522		5.176	Y	NP	4.091	3.964	Y	P
**L320Y**	4.623		4.377	Y	NP	6.148	5.375	N	NP
**L320C**	6.872		4.146	Y	NP	4.129	3.799	Y	P

FLIP refers to whether the sugar portion of the substrate has turned 180° within the active site. Productive (P) or non-productive (NP) refers to the overall binding mode of the predicted pose. PDB codes for docking HGALE/UDP-Glcp (1EK6), EcGALE/UDP-Glcp (1XEL), HGALE/UDP-GlcpNAc (1HZJ), EcGALE/UDP-GlcpNAc (1LRJ).

While productive binding modes were predicted for HGALE (1JZJ) with each member of the larger UDP-Glc*p*NAc/UDP-Gal*p*NAc pair, the equivalent docking within EcGALE (1LRJ) lead to non-productive binding modes. These results agree with the reported N-acetylated interconversion ability for the larger HGALE active site and the lack of activity by EcGALE due to the smaller active site. The AnGALE docking results for the larger substrate pair also follow the observed experimental activity. Docking within wild-type AnGALE showed a non-productive binding mode for UDP-Glc*p*NAc and a potentially active pose for UDP-Gal*p*NAc. Observance of a productive binding mode could be attributable to the active site accommodating the larger substrate while not being of sufficient size to allow for the rotation of the substrate during catalysis. In this context, the docking results seem to support the observed AnGALE inhibition by the N-acetylated derivatives. Non-productive binding modes were also identified for UDP-Gal*p*NAc and UDP-Glc*p*NAc within the AnGALE L320Y modelled mutant which exhibits a smaller active site. The weak inhibition of the L320Y mutant by the UDP-Gal*p*NAc is supported by these findings. Lastly, the L320C modelled mutant, by virtue of its experimentally observed activity, was expected to show productive binding modes for the UDP-Glc*p*NAc/UDP-Gal*p*NAc pair. Interestingly, although a productive pose was observed for UDP-Gal*p*NAc, initial efforts to dock UDP-Glc*p*NAc resulted exclusively in non-productive binding modes. The lack of a productive pose could be explained in light of the EcGALE Y299C mutant analysis which revealed a larger active site can result in decreased activity as the substrate may be presented with additional binding options. Closer inspection of the active sites for AnGALE and HGALE, which the L320C mutant was designed to mimic, revealed a larger residue, Y218 in AnGALE (N206 in HGALE), could be sterically preventing the productive binding mode. In fact, introduction of Y218 flexibility into the docking procedure resulted in the side chain shifting to accommodate the N-acetyl group of UDP-Glc*p*NAc which ultimately allowed for a productive binding mode with d_Y-S_ of 5.388 Å and d_C4-C4_ of 4.576 Å. These results are in line with recent computational work performed on both HGALE and EcGALE which showed substrate specificity is not only influenced by size, but also by protein flexibility near the active site [74]. Comparison of molecular dynamics simulations for these epimerases revealed increased flexibility of an active site loop in HGALE which could help explain the additional specificity towards NAc-derivatized substrates.

## Conclusions

The structural analysis of AnGALE complexed with UDP-Glc*p* allowed for a detailed comparison with the Human epimerase. Given the similarities between the structures, and more specifically, the near identical composition and orientation of active site residues along with the preservation of catalytically important contacts, implies that the mechanism for AnUGM is likely the same as that established for HGALE. The mechanistic similarity was also supported by the lack of activity for AnGALE mutants of the conserved Y-X-X-X-K catalytic motif, Y156F and K160V.

The overall activity profile for wild-type AnGALE towards UDP-Glc*p*/UDP-Gal*p* and UDP-Glc*p*NAc/UDP-Gal*p*NAc interconversion more closely resembles that of EcGALE. The results are in line with what was expected given the relative size of the AnGALE L320 gatekeeper as compared to HGALE and EcGALE. The *K*_m_ and *k*_cat_ for UDP-Gal*p* interconversion for wild-type AnGALE were determined to be 0.11 mM and 12.8 s^-1^, respectively. Additionally, our studies have shown that the ability of AnGALE to interconvert UDP-Glc*p*NAc/UDP-Gal*p*NAc can be activated through a single point mutation of the gatekeeper residue, namely L320C. Similar findings were reported previously for EcGALE through the equivalent Y299C point mutation.

Docking studies of AnGALE with substrates were conducted to further probe active site affinity towards both substrate pairs. Reproduction of observed binding modes validated the GALE docking procedure and criteria by which predicted binding poses were judged to be productive were also established. Overall, the docking results showed general agreement with the experimentally observed activity profiles for each of the epimerases investigated. Minor deviations within the docking predictions from what was anticipated for AnGALE were rationalized in terms of the active site allowing substrate binding while preventing the rotation required for substrate turnover. In this sense, the docking results also agree with the general findings of the AnGALE inhibition experiments.
